# Fatal and Near-Fatal Non-allergic Reactions in Patients with Underlying Cardiac Disease Receiving Benzathine Penicillin G in Israel and Switzerland

**DOI:** 10.3389/fphar.2017.00843

**Published:** 2017-11-21

**Authors:** Matitiahu Berkovitch, Liat Ashkenazi-Hoffnung, Ilan Youngster, Dotan Shaniv, Dorit Dil-Nahlieli, Einat Gorelik, Martina Schäublin, Rudolf Stoller, Nour Karra, Eyal Schwartzberg

**Affiliations:** ^1^Clinical Pharmacology Unit, Assaf Harofeh Medical Center, Zerifin, Israel; ^2^Sackler Faculty of Medicine, Tel-Aviv University, Tel-Aviv, Israel; ^3^Department of Pediatrics B, Schneider Children's Medical Center of Israel, Petah-Tikva, Israel; ^4^Pharmacovigilance and Risk Management Department, Israeli Ministry of Health, Jerusalem, Israel; ^5^Division for safety of medicines, Swissmedic, Swiss Agency for Therapeutic Products, Bern, Switzerland; ^6^Pharmaceutical Division, Israeli Ministry of Health, Jerusalem, Israel; ^7^School of Pharmacy, Ben Gurion University of the Negev, Beer-Sheba, Israel

**Keywords:** penicillin, death, case-series, non-allergic, adverse-events

## Abstract

Benzathine Penicillin G (BPG) is commonly used for treatment of penicillin-susceptible infections and secondary prevention of rheumatic fever. Death following administration of BPG is extremely rare—only a handful of cases have been described in the literature since the 1950's. In this case series from Israel and Switzerland, we describe nine cases of serious adverse reactions—six fatal reactions and three near-fatalities—occurring within minutes of receiving intramuscular BPG. Allergic reactions or faulty administration were not implicated in any of the cases; however, all patients had cardiac risk factors. This case series describes a relatively rare risk that should be borne in mind when prescribing BPG.

**What is Already Known on the Subject:**

- Benzathine penicillin G (BPG) is a very common parenterally-administered antibiotic that should only be given by the intramuscular route.
- Inadvertent administration by the intravenous route has been associated with cardiorespiratory arrest and death.
- Fatal reactions to BPG have been rarely reported since the 1950's.

**What this Study Adds:**

- Fatal non-allergic reactions to BPG may occur in both adults and children after intramuscular administration.
- Fatal non-allergic reactions to BPG may occur as quickly as a few minutes following administration.
- This report should encourage healthcare professionals to administer BPG only when clearly indicated and to be prepared for life-threatening situations.

## Introduction

Benzathine Penicillin G (BPG) is commonly used for treatment of penicillin-susceptible infections such as syphilis, erysipelas, and group A streptococcal disease. Another common indication for use is the secondary prevention of rheumatic fever. BPG is a tetrahydrate compound of penicillin G and dibenzylethylenediamine, intended for deep intramuscular (IM) injection. Its prominent feature is the prolonged duration of action compared to other parenteral penicillins, resulting from its unique composition. The formulation has low water solubility and is slowly hydrolyzed within the muscular compartment, from which the penicillin G is gradually released into the systemic circulation over 2–4 weeks (Wyber et al., 2013). BPG is generally reserved for treatment of infections caused by pathogens that are highly susceptible to penicillin, since the serum levels are relatively low due to the unique release kinetics. Nevertheless, it is listed in the World Health Organization's List of Essential Medicines (WHO, 2015).

Despite BPG's favorable safety profile (Galvao et al., 2013), a few severe adverse events have been reported since it was first marketed in 1954. The well-known adverse event of BPG administration is related to the risk of immediate-type hypersensitivity reactions to penicillin manifesting as anaphylactic shock and possibly death (Galvao et al., 2013). However, less familiar non-allergic reactions have also been described. According to a systematic review by Galvao et al. (2013), four cases of death in the general population on account of BPG administration have been reported between 1954 and 2012. Another report by Steigmann and Suker (1962) described three more cases, unaccounted for in the review by Galvao et al. In addition, the medication is currently marketed with a black-box warning regarding intravenous administration, based on several reports of inadvertent IV administration of BPG that resulted in cardiorespiratory arrest and death (Steigmann and Suker, 1962; Levetan et al., 1988; Gerber and Apseloff, 2002). The most likely pathophysiologic explanation for these fatalities is that the BPG compound is a crystalline powder that may cause direct damage when injected into a blood vessel, possibly eliciting vascular spasm and subsequent occlusion by the large crystals of the penicillin salt (Gerber and Apseloff, 2002). BPG also carries a warning regarding impairment of renal function—since BPG is excreted mainly by the kidneys, patients with suboptimal renal function may have a relatively higher risk for crystal formation and subsequent tubular damage (1991).

On January 2015, the Israeli Ministry of Health's Pharmacovigilance Department received three reports of deaths temporally associated with an IM administration of BPG. Subsequent investigations revealed one additional death and two additional cases of severe, near-fatal, adverse reactions temporally associated with IM administration of BPG. Communications with the Swiss Agency for Therapeutic Products, Swissmedic, revealed two fatalities and one severe reaction associated with the administration of BPG that were reported in Switzerland. All patients in both countries had underlying cardiac risk factors. As a result of these cases, and the fact that no plausible explanation other than direct cardiac toxicity was suggested, in February 2015 the Israeli Ministry of Health issued an official safety recall, stating that preparations containing BPG should not be administered to patients until further notice, unless no equivalent therapeutic alternative is available^1^.

This case series aims to present these non-allergic fatal and near-fatal consequences of BPG administration to patients with cardiac disease in Israel and Switzerland and discuss the unique nature of such reactions, with the aim of raising the awareness to the potential less familiar complications of BPG administration.

## Description of the case reports

Data of all 9 patients is summarized in Table 1.

**Table 1:** Clinical data of nine reported cases of severe non-allergic adverse reactions associated with Benzathine Penicillin G.

**Case**	**Age (years)**	**Gender**	**Indication for BPG administration**	**Dose (MU)**	**Co-administration of lidocaine**	**Time from BPG administration to onset of event**	**Background diagnoses**	**Description of event**	**Outcome**
1	77	F	Recurrent erysipelas	1.2	Yes (1%)	< 1 min	DM2 HTN AF PVD CHF Tricuspid regurgitation Pulmonary HTN	Loss of consciousness, asystole No accompanying symptoms	Death
2	70	F	Recurrent erysipelas	2.4 (in two doses)	Yes (1%)	Seconds after 2nd dose	CHF AF Post-aortic valve replacement	Loss of consciousness, asystole No accompanying symptoms	Death
3	91	F	Recurrent erysipelas	1.2	Yes (1%)	Seconds	IHD COPD LVH HTN Dementia AF	Loss of consciousness, asystole No accompanying symptoms	Death
4	66	M	Recurrent erysipelas	2.4 (in two doses)	Yes (1%)	Seconds after 2nd dose	Lymphoma HTN AF CHF Aortic regurgitation	Loss of consciousness, cyanosis No accompanying symptoms	Death
5	93	M	Recurrent erysipelas	1.2	Yes (1.5%)	During injection	DM1 IHD CHF HTN PVD CRF	Loss of consciousness, cyanosis	Underwent CPR. Subsequently recovered
6	81	F	Recurrent erysipelas	1.2	Yes (1%)	1 h	HTN Dementia CHF IHD	Loss of consciousness, regained spontaneously. Subsequently complained of nausea, cold sweat	Patient recovered
7	10	M	Prevention of RF	1.2	No	A few minutes	Severe mitral insufficiency Chronic endocarditis Post-RF	Cardio-respiratory arrest	Death
8	12	M	Prevention of RF	0.6	No	Seconds	Severe mitral insufficiency AF Post-RF	Loss of consciousness, asystole	Death (Initially regained cardiac activity, died 2 days later)
9	87	F	Sinusitis	2.4	No	Seconds	DM2 HTN AF Osteoporosis	Loss of consciousness, vomiting, diaphoresis	Patient recovered

*Cases 1–6, reported from Israel, cases 7–9 reported from Switzerland*.

*AF, atrial fibrillation; CHF, congestive heart failure; COPD, chronic obstructive pulmonary disease; CRF, chronic renal failure; DM, diabetes mellitus; HTN, hypertension; IHD, ischemic heart disease; LVH, left ventricular hypertrophy; PVD, peripheral vascular disease; RF, rheumatic fever; CPR, Cardiopulmonary Resuscitation*.

Nine patients, 5 females and 4 males, who experienced serious non-allergic reactions related to BPG administration, were reported. Indications for BPG included recurrent erysipelas (6 cases), prevention of rheumatic fever (2 cases), and sinusitis (1 case). Age ranged from 10 to 93 years. Dosage of BPG ranged from 0.6 to 2.4 Million Units. In all cases but one, BPG was routinely administered many times before the reaction.

In close temporal relationship [during (*n* = 1); immediately after (*n* = 7); 1 h after (*n* = 1)] to the intramuscular BPG application, the patients developed similar reactions with loss of consciousness and in most cases asystole/cardiorespiratory arrest. Typical allergic symptoms like urticaria or angioedema were missing in all cases. Three patients recovered, while in six cases the outcome was fatal despite intense resuscitation efforts. All cases had underlying cardiac conditions such as, congestive heart failure, atrial fibrillation, ischemic heart disease, ventricular hypertrophy, and severe mitral insufficiency.

Applying the WHO-UMC system for standardized case causality assessment^2^, the resulting assessment is “Possible” in all cases, i.e., the event was with reasonable time relationship to drug intake, could also be explained by disease or other drugs, and information on drug withdrawal may be lacking or unclear.

## Discussion

In the current report, we describe nine cases of severe non-allergic reactions associated with IM injection of BPG, with six fatalities. Few reports of fatal reactions to IM injections of BPG have been published previously, and the possible mechanism remains obscure (Steigmann and Suker, 1962). In a prospective study, where BPG was administered to 1,790 patients as a prophylaxis for rheumatic fever, the fatality rate was 0.05% (0.31/10,000 injections), however, all deaths were attributed to hypersensitivity reactions (1991), which are implausible in our series. Hypersensitivity reactions to penicillin may include pruritus, urticaria, flushing, wheezing, laryngeal edema, angioedema, abdominal distress, and hypotension (Weiss and Adkinson, 1988; 1991). None of these typical signs and symptoms were described in our case series. Furthermore, in the vast majority (8/9), the patients had been repeatedly exposed to BPG prior to the adverse event or death. In 1962, Steigmann and Suker published three cases of fatalities associated with injections of BPG (Steigmann and Suker, 1962). It should be noted that all three patients had cardiomegaly and a history of cardiac decompensation. Similarly, all patients included in our report had underlying cardiovascular disease, although they were in a stable clinical condition prior to the administration of the BPG injection.

In the cases from Israel, the injected BPG was from different batches and manufacturers, and further investigation of the vials, contents and related documentation did not reveal any quality issues. In all instances, the BPG was diluted with a small amount of lidocaine (1–1.5%) to minimize injection-associated pain and proper procedures to avoid intravascular injection of the content were followed (i.e., trying to aspirate blood prior to injection). Lidocaine Hydrochloride (Lignocaine HCl) 1% is an isotonic sodium chloride solution, which contains no antibacterial preservatives. Lidocaine hydrochloride is metabolized mainly in the liver and excreted via the kidneys, mainly in the form of various metabolites. Lidocaine hydrochloride solutions are indicated for the production of local or regional anesthesia. The individual dose of a lidocaine hydrochloride solution, should be such that it is kept below 300 mg and, in any case, should not exceed 4.5 mg/kg body weight^3^. For a patient weighing 70 kg, the maximal recommended volume of lidocaine solution is around 30 ml. The patients described in our case series received a maximal dose of 3 ml of 1% lidocaine solution, equal to 30 mg of lidocaine, which is significantly below the maximal recommended dose (0.4 mg/kg body weight of a 70 kg patient). Furthermore, lidocaine was not added to the injections administered in our case series from Switzerland. Therefore, the co-administration of lidocaine is an unlikely explanation for the reactions described.

Among the three cases from Switzerland, two cases involved injections from a single batch, but no quality issues were detected by the investigating health authorities.

Unfortunately, no post-mortem data was available for any of the described cases.

Accidental IV administration of BPG cannot be ruled out, but in all cases the injection was administered by experienced personnel and was reported as deep IM delivery. Intravascular passage of micro-crystals (which may provoke micro-emboli) after IM administration is possible and has been hypothesized to cause dyspnea, cyanosis and neuropsychiatric symptoms (Hoigné Syndrome). However, this has been described mainly with use of procaine-penicillin and furthermore, the rapid onset and prominent cardiac conditions in our cases make this explanation unlikely.

It should be pointed out that these adverse reactions do not seem to be age-associated. In the aforementioned cases, the patients' ages ranged from very young to very old. The cases described by Steigmann and Suker (1962) complete the middle range with a 19 years old patient, 49 years old patient, and 31 years old patient. This may exclude the age factor as a potential risk for adverse effects following IM injections of BPG.

Finally, we sought to examine the possible involvement of the other constituent of BPG, namely, dibenzylethylenediamine, in the adverse reaction mechanism. This substance is added to the formulation to prolong penicillin's half-life, as it significantly lowers water solubility and necessitates hydrolyzation for the release of the active penicillin G from the formulation. No clinical information regarding possible cardiac and/or respiratory toxicity of dibenzylethylenediamine has been found in the published literature, possibly because this substance only serves as a pharmacokinetic modulator and has no medical use of its own.

The WHO-UMC system for standardized case causality assessment includes 6 categories: certain, probable/likely, possible, unlikely, conditional/unclassified, and unassessable/unclassified^2^. Applying the WHO-UMC system, the resulting assessment in all cases was “Possible”, i.e., the event was with reasonable time relationship to drug intake (<1 min after the BCG administration in the majority of cases), could also be explained by disease or other drugs, and information on drug withdrawal may be lacking or unclear.

All patients described in our case series, including the children, had an underlying cardiac condition, which might have contributed to the serious events and deaths.

In summary, we reported nine cases of severe non-allergic events associated with injections of BPG in two countries. These reports, combined with a limited number of cases of similar nature reported in the past, reinforce the notion that BPG IM should be used with caution and only when indicated.

## Author contributions

MB, IY, DS, DD-N, EG, MS, and RS have collected the data, performed the investigation and literature search, and wrote the manuscript in collaboration. LA-H, NK, and ES have reviewed the manuscript and suggested valuable comments, which improved the quality of the manuscript.

### Conflict of interest statement

The authors declare that the research was conducted in the absence of any commercial or financial relationships that could be construed as a potential conflict of interest.

## Footnotes

^1^Israeli Ministry of Health Announcement Regarding BPG (2015). Available online at: http://www.health.gov.il/NewsAndEvents/SpokemanMesseges/Pages/15012015_4.aspx (Accessed 3 May, 2016).

^2^The use of the WHO-UMC system for standardized case causality assessment World Health Organization (WHO) – Uppsala Monitoring Center. Available online at: https://who-umc.org/media/2768/standardised-case-causality-assessment.pdf.

^3^Drug monograph: Esracain Injection 1%: 019 20 21029 00 Reg. no. Esracain Injection 2%: 020 82 21028 00 Rafa Laboratories, POB 405, Jerusalem 91003, The format and content of this document have been approved by the Ministry of Health in January 2007.
